# Table tennis motion recognition based on the bat trajectory using varying-length-input convolution neural networks

**DOI:** 10.1038/s41598-024-54150-5

**Published:** 2024-02-12

**Authors:** Jun Zhang, Yuanshi Ren, Liyue Lin, Yu Xing, Jie Ren

**Affiliations:** 1 School of Exercise and Health, Shanghai University of Sport, Shanghai, 200438 China; 2 China Table Tennis College, Shanghai University of Sport, Shanghai, 200438 China; 3 School of Psychology, Shanghai University of Sport, Shanghai, 200438 China; 4 School of Sport Communication and Information Technology, Shandong Sport University, Jinan, 250102 Shandong China

**Keywords:** Computational models, Data acquisition, Data processing

## Abstract

Action recognition has been applied in fields such as smart homes, gaming, traffic management, and security monitoring. Motion recognition is helpful for biomechanical analysis, auxiliary training systems, table tennis robots, motion-sensing games, virtual reality and other fields. In our study, we collected data on table tennis skill motion, created the TTMD6 dataset, and analyzed the characteristics of table tennis paddle trajectories. We propose a motion recognition algorithm to recognize paddle trajectories. Other research has used multijoint data to identify actions, while we use only the paddle trajectory to recognize table tennis skill motions, accelerating the speed of motion recognition. Therefore, it is feasible to use paddle trajectories to recognize table tennis skill motions.

## Introduction

A time series is a collection of data points arranged in chronological order. Since time series data can reflect dynamic changes, feature extraction and extraction algorithms for time series data have become important research topics in many fields, such as biomedical engineering^1,2^, speech detection^3^, finance and other fields^4,5^. With the successful application of machine learning and deep learning^6,7^ in feature recognition, the problem of feature extraction based on time series has new vitality. Traditional methods usually use dynamic time warping (DTW)^8^ or shapelet transforms^9^ to extract the discriminant features of the original time series. These two methods separate the feature extraction module from the classification module, which limits algorithm accuracy. In addition, most of the existing methods do not solve the problem that time series may have different time scales.

Dynamic time warping (DTW) involves expanding and shortening two time series to obtain two time series of the same length. For example, in speech recognition, because the time series of the source speech and the target speech to be processed are different lengths, DTW can adjust the time series of different lengths to the same size^10^.

The shapelet transform can be used for time series classification^11^ and data enhancement^12^. Cui et al.^12^ applied the shapelet transformation method to all time series in a given dataset when studying the multiscale convolutional neural network model and regarded all data slices as independent training instances.

The weaknesses and advantages of the four methods can be found in Table 1.

**Table 1:** The weaknesses and advantages of the four methods.

No	Method	Weaknesses and advantages
1	Truncation and zero filling	Easy to implement, but can cause data distortion
2	Dynamic time warping	A source object is needed, which is not suitable for motion recognition
3	Shapelets	Time series of different lengths can be sliced to the same length using window slicing. However, the size of the window slicing is crucial to the recognition result
4	Varying-length-input	Data preprocessing is divided into three cases, and our method preserves the original characteristics of the data to the greatest extent

The duration of table tennis motion differs due to differences in athlete motion ability and task goals, which can also cause problems with different durations of table tennis motion recognition. To solve the time series and data volume problems, we use an infrared motion capture system to collect the time series of six popular motions (forehand attack, forehand drive, forehand push, backhand attack, backhand drive, and backhand push) in table tennis and create the time series dataset TTMD6 (Table Tennis MOCAP Dataset). The TTMD6 dataset contains 9,000 strokes of 6 table tennis motions, independent human joint data and independent paddle center of gravity data. We used MATLAB to create a skeletal animation of the movement trajectory of the paddles, recruited table tennis players for recognition and achieved a recognition accuracy of 92.63%. In this study, we propose a varying-length-input convolutional neural network framework to recognize paddle trajectories and obtain a recognition accuracy of 99.78%, which is much greater than that of athletes.

## Convolutional neural networks

Neural networks are important methods for studying artificial intelligence. At present, one of the most popular neural networks is the convolutional neural network (CNN)^13–15^. CNNs were first used in the ImageNet Large-scale Visual Identification Challenge (LSVRC) in 2012 by Krizhevsky et al.^16^. By adopting ReLU+dropout technology, researchers achieved the best classification results at that time (the network structure called AlexNet), which made CNNs increasingly valuable to researchers. Compared with AlexNet, Szegedy et al.^15^ greatly increased the depth of CNNs and proposed a CNN structure with more than 20 layers (the model called GoogLeNet). The main advantage of this structure is that it improves the utilization of computing resources and has greater accuracy.

The AlexNet, GoogLeNet and VGG models all achieved good results in the ImageNet competition. However, they can accept only a fixed-size input. In fact, the convolutional layer of a CNN does not require a fixed-size input; it can generate feature surfaces of arbitrary size, but its fully connected layer requires a fixed-length input, so the restriction of consistent input length of CNNs stems from its fully connected layer^17^. The input image needs to be cropped or scaled to obtain a fixed-size input, but such a transformation will destroy the aspect ratio and complete the information of the input image to affect the recognition accuracy. He et al.^17^ proposed an SPPNet model in which a spatial pyramid pooling (SPP) layer is added between the last convolutional layer and the first fully connected layer of CNNs. The SPP layer can make inputs of different sizes produce outputs of the same size, which breaks the restriction that the input of the CNN model is fixed in size.

In the^6,7^ network model with very deep layers, there are gradient diffusion problems and degradation problems. Batch normalization (BN) is an effective method for solving the gradient diffusion problem^18^. He et al.^14^ proposed residual networks (ResNets) to solve the degradation problem.

As AIs have developed, CNNs have been used in speech recognition^19^, face recognition^20^, image recognition^14^, motion analysis^7^, motion analysis^21^, image segmentation^22^, natural language processing^23^ and EEG analysis^5^.

## Table tennis MOCAP dataset

### Participants

The thirty professional table tennis players (17 males and 13 females) who volunteered to participate in the data acquisition were all undergraduates of the China Table Tennis College of Shanghai University of Sport, and they were all national division I athletes, national division II athletes, or national master sportsmen who were right-handed. At least three months before the experiment, all participants had no injuries or deformities to the lower extremities. Moreover, they were required not to consume any coffee or food with related additives, such as caffeine, prior to 6 h of the experiment and signed a written informed consent. The experiment was approved by the Ethics Committee of Shanghai University of Sport (ethics approval reference: 102772019RT030). All methods were performed in accordance with ethical approval.

### Apparatus

The experiment was carried out on a standard-sized table. The table tennis ball was sent to the participants by a ball projection machine (Y&T® V-989H, Zhongshan, China) on the opposite side of the table. The serving frequency was set at 30 Hz, and the ball speed was 6.5 m/s. All the subjects used the same paddle to complete the task during the experiment. All motion was captured by both the full HD camera and the MOCAP system. The full HD camera is situated behind the ball projection machine. The camera is 2.30 m above the ground, and the sampling frequency is 25 Hz, which is used to record the frontal shot of the participant. The MOCAP system (CMTractor 2.0; Shanghai Qingtong Vision Co., Ltd., China) consists of 20 infrared cameras fixed on the ceiling of the laboratory with a capture frequency of 120 Hz. A camera array was used to track the 3D positions of 38 reflective marks placed on the participants’ bodies.

Each participant needed to perform six skill motions. The experiment was divided into two stages: 1) the participant's adaptation stage before the experiment and 2) the motion capture stage. Before the test, the participants were told that the table tennis ball would be launched from the ball projection machine on the opposite side and that they would have to hit the cross-court ball. After the adaptation stage (5 serves), the participants complete the data acquisition of 55 shots without further instructions.

**Figure 1:**
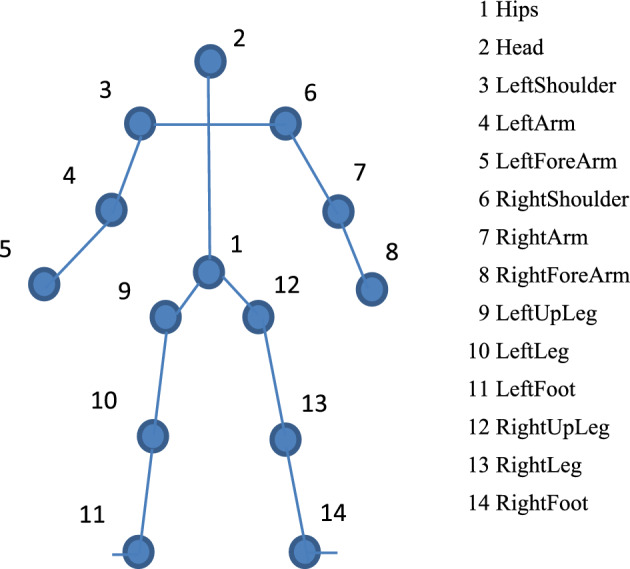
Skeleton diagram of 14 joints.

The 14-joint skeleton diagram is similar to the 15-joint human body model of S. Litvak^24^. The only difference is that the former does not have the shoulder center, which is located between the left shoulder and the right shoulder, compared with the latter. The shoulder center can be calculated. We collect 14 joint time series data and the center of gravity of the table tennis paddle to construct the TTMD6 dataset. The 14 joints used by TTMD6 are shown in Fig. 1.

### Data processing

The complete table tennis motion includes four stages: backward swing, stroke, follow-through, and recovery. The motion capture system recorded 55 shots of the participants at a time, and the 55 shots were saved in a file. Each file saves nearly 20,000 frames of data, and a single motion needs to be extracted. First, the analyzer software of the motion capture system was used to manually mark the starting frame of each motion, and then the motion extraction tool written in C# was used to extract a single motion. The motion visualization tool written in MATLAB is an alternative tool to the analyzer software of the motion capture system. This tool can display motions in the form of skeleton diagrams.

### Athletes recognize table tennis motions

Table tennis is one of the most popular paddle sports in the world; however, with more than 100 years of development, many different types of skill motions, such as attack, drive, long push, short push, loop, push-and-block, drop shot, off-table chop, smash, and lift, have been developed^25^. Each of these pongs hit a different position on the incoming ping pong. Accurate recognition of these motion patterns is highly important for the development of table tennis robots, daily training of athletes and somatosensory games.

Acquiring table tennis skills depends on the guidance of coaches. Experienced coaches can determine the type of motion according to the type of body movement performed by the athlete. Athlete recognition was introduced to test the recognition accuracy of professional athletes. Our research purpose was to identify an algorithm that exceeds the recognition accuracy of traditional methods for athletes to prepare for the next step of constructing an auxiliary training system.

**Figure 2:**
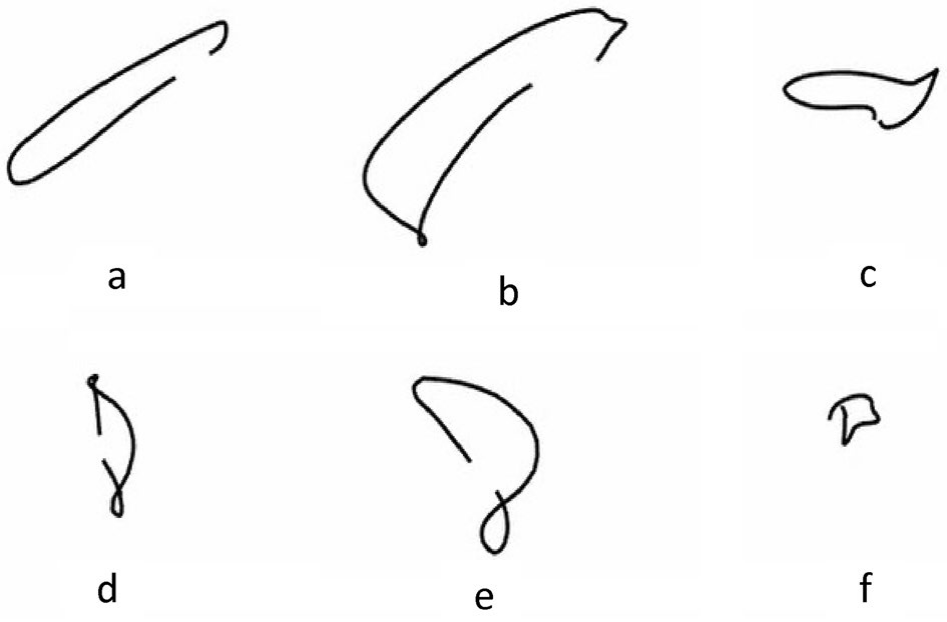
Bat trajectory of table tennis motion (**a**) forehand attack (**b**) forehand drive (**c**) forehand push (**d**) backhand attack (**e**) backhand drive (**f**) backhand push.

Unlike other motion recognition methods, in table tennis, players hit table tennis with a paddle. The movement trajectory of a paddle is the embodiment of the coordination of all joints, and an athlete can control the incoming ping pong through the movement of the paddle. The paddle was located on the extension line of the distal end of the upper limb. Compared with the shoulder, elbow and wrist, the paddle has a greater range of motion and a faster movement speed, which can better reflect the movement characteristics of table tennis. The paddle trajectories of the six skill motions are shown in Fig. 2. Experienced coaches and athletes can predict the type of motion, the trajectory of the ping pong ball and its landing point from the movements of the opponent’s body and the trajectory of the paddle. The purpose of this experiment was to demonstrate the feasibility of motion recognition through a paddle trajectory.

**Figure 3:**
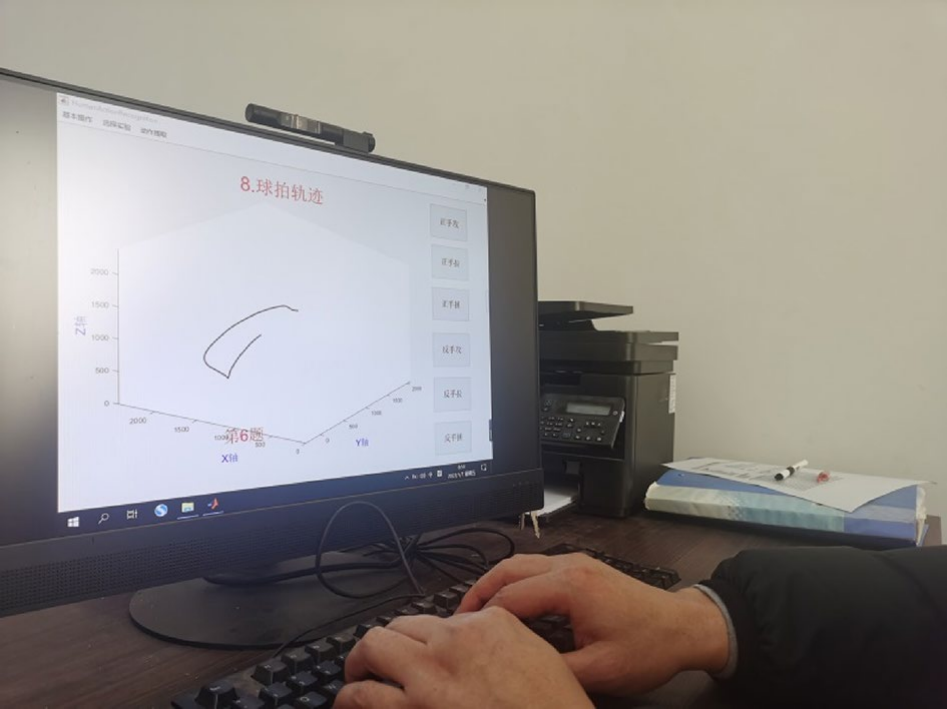
The athlete recognizes bat trajectory on PC.

We selected 30 motions (5 for each skill motion) and used MATLAB software (version 2018A; MathWorks, Inc., USA) to generate animations, and the interval between motions was 2 s. Q, W, E, I, O, and P on the keyboard correspond to forehand attack, forehand drive, forehand push, backhand attack, backhand drive, and backhand push, respectively. After recognizing the motion, the athletes pressed the key on the keyboard as quickly as possible and entered the recognition result. The program records the recognition result of the athletes' input and the time it takes for them to recognize the motion (the first frame of the motion appears until the participant presses the key). We recruited 40 professional table tennis athletes from Shanghai University of Sport to participate in this study. Figure 3 shows the athlete’s recognition of the paddle trajectory on the computer.

### Varying-length-input convolution neural networks

The basic CNN structure consists of an input layer, a convolutional layer, a pooling layer, a fully connected layer and an output layer. Generally, several convolutional layers and pooling layers are contained, convolutional layers and pooling layers are set alternately, and then a convolutional layer is connected after the pooling layer^26^.

The convolutional layer of CNNs does not need a fixed-size input, but its fully connected layer does. Therefore, the limitation that the input sizes of CNNs must be consistent is due to the fully connected layer used^17^. The input image needs to be cropped or scaled in the field of image recognition to obtain a fixed-size input; however, such a transformation will destroy the aspect ratio and complete information of the image, thereby affecting the recognition accuracy. He et al.^17^ proposed an SPP-net model, which adds a spatial pyramid pooling (SPP) layer between the last convolutional layer and the first fully connected CNN layer. The SPP layer can make different sizes of CNN inputs produce the same size of output, which breaks the previous limit of CNN model input fixed, and the improved CNN model has a faster training speed.

**Figure 4:**
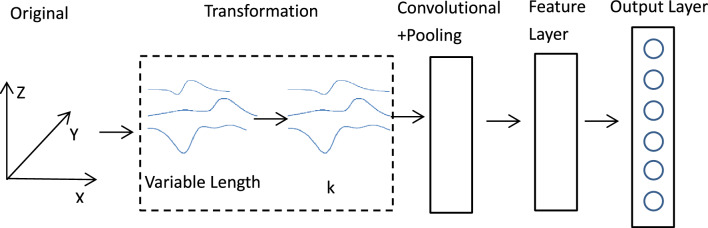
Over architecture for 3D time series classification.

The duration of table tennis motion varies due to the athlete's ability to perform the motion and the target of the task. When training the model, DTW^10,27^ and Shapelets^9,28^ are used to truncate and adjust the time series to a fixed length, but such transformation will destroy the complete information of the time series. To solve these problems, we design a time series transformation layer in the input layer and convolution layer and integrate the transformation layer and CNN into a framework, as shown in Fig. 4.

Interpolation is a common method for data preprocessing. The most commonly used interpolation methods include nearest neighbor interpolation, linear interpolation and spline interpolation. Spline interpolation is a commonly used interpolation method for obtaining smooth curves. The cubic spline method is one of the most widely used interpolation methods. The basic idea of cubic spline interpolation is to divide n intervals within [a, b] and perform cubic spline interpolation fitting for each interval to generate a smoother curve.

In the time series transformation, l represents the actual length of the time series (the file name contains the actual length information), and k represents the length of the transformed time series. When dealing with a time series, we can divide it into three cases: k > l, k = l, and k < l.

When l > k, we downsample the raw data to a fixed length. Assuming that the time series T = {t_1_…,t_n_}, the downsampling ratio is l/k, the ith element of the transformed time series, ti = x[**⌈**i*l/k **⌉**], 0 <  = i < k, and **⌈·⌉** denoting the operation of the ceiling.

When l = k, there is no change in the time series.

When l < k, we use cubic spline interpolation^29^ to interpolate the time series. Formula 1 is a mathematical formula describing cubic spline interpolation. In this article, the cubic spline interpolation is implemented using Python's SciPy toolkit.

1 $$\left\{\begin{array}{c}S\left(x\right)\epsilon {C}^{2}[a,b]\\ {S}_{({x}_{i})}={y}_{i}\\ S\left(x\right)={a}_{3}{x}^{3}+{a}_{2}{x}^{2}+{a}_{1}x+{a}_{0}\end{array}\right.$$

In the extreme case, if k is less than the length of the shortest time series, all the time series are downsampled only. The time series length of a group of motions has a range. For example, a participant needs at most 212 frames and at least 169 frames to complete a forehand attack. When k < 169, we do not need to consider the first two cases but only the third case. However, if k is too small, it will affect the recognition accuracy. When all the time series are of the same length, the neural network can be trained.

## Discussion

To produce the TTMD6 dataset, we recruited 30 professional table tennis athletes from Shanghai University of Sport to participate in this test. The age range of the 30 participants was 14 to 24 years, which allowed more realistic changes in motion quality to occur. Since the infrared camera is fixed to the ceiling and the TTMD6 dataset is acquired in the laboratory, we evaluate environmental instability under various background conditions. The large differences in views and participants make cross-evaluation possible.

K-fold cross-validation^8^ is a strategy for dividing datasets. When the dataset is small, using all the data to train the model will easily lead to overfitting. K-fold cross-validation can solve the problem of overfitting the small dataset training model to a certain extent. K-fold cross-validation can also be used for model evaluation and model selection. We used fivefold cross-validation to divide the TTMD6 dataset and adopted a CNN to test the stability of TTMD6, as shown in Table 2.

**Table 2:** Results of fivefold cross-validation.

No	Part1	Part2	Part3	Part4	Part5	Accuracy
1	Train	Train	Train	Train	Test	96.16%
2	Train	Train	Train	Test	Train	98.44%
3	Train	Train	Test	Train	Train	98.94%
4	Train	Test	Train	Train	Train	99.56%
5	Test	Train	Train	Train	Train	95.83%

The stability of the TTMD6 dataset was tested by fivefold cross-validation. In our research, we divided the whole dataset into 5 parts, one of which was used as the test set and the other as the training set. The training set was used to train the model, and the test set was used to test the trained model. The whole process was performed five times. As shown in Table 2, the results of the five experiments are relatively stable, with the lowest accuracy rate of 95.83% and an average recognition accuracy rate of 98.38%. The test results show that the TTMD6 dataset has good stability.

Compared to body joints, the paddle is located on the extension line of the distal end of the upper limb, and the movement trajectory of the paddle can more effectively reflect the characteristics of each skill motion. Experienced coaches and athletes can predict the motion type, trajectory and landing point of the ping pong through the opponent’s body movements and trajectory. We recruited 40 professional table tennis athletes from Shanghai University of Sport to perform the animation of paddle trajectories. Table 3 shows the confusion matrix of athletes who recognized table tennis motions.

**Table 3:** The confusion matrix of participants recognizing table tennis motions.

Motion	FA	FD	FP	BA	BD	BP
FA	1332	160	0	8	0	0
FD	63	1429	0	0	8	0
FP	14	14	1464	0	0	8
FA	0	8	0	1218	250	24
BD	8	21	14	48	1409	0
BP	0	0	10	0	8	1482

A confusion matrix, also known as an error matrix, is a standard format for evaluating accuracy and is expressed in the form of a matrix with n rows and n columns. Each column of the confusion matrix represents the predicted category, and the values in each column represent the number of data points predicted as the category. Each row represents the true category of the data, and the total number of data points in each row represents the number of data points in that category. Table 3 shows that the recognition accuracy of forehand push and backhand push is the highest, 97.7% and 98.6%, respectively, followed by forehand drive and backhand drive, and the lowest recognition accuracy is for forehand and backhand attacks. As it is easy to confuse forehand attacks with forehand drives and backhand attacks with backhand drives, most of the recognition errors made by athletes are mainly due to confusion from attacks and drives whose essentials are familiar. Figure 3 shows that the strength, speed, and movement range of the drives are greater than those of the attack. A drive with a slightly smaller movement range is easily recognized as an attack, while an attack with a slightly larger movement range is easily recognized as an attack. The average accuracy of athlete recognition by paddle trajectory was 92.63%. Therefore, it is feasible to use the paddle trajectory for motion recognition. Using the paddle trajectory to train the neural network greatly reduces the quantity of data and the time required to train the model.

With the development of AIs, human action recognition has made many breakthroughs in recent years. However, there is not much work on table tennis motion recognition, and the methods are mainly integrated into traditional machine learning. In addition, traditional CNNs generally use truncation and zero-filling methods to process time series of different lengths, which leads to data distortion and affects the recognition effect of the algorithm. The method we propose adjusts time series of different lengths to the same length, and the recognition accuracy of this method is higher than that of traditional processing methods. To solve the problems of time series length and data quantity, we use paddle trajectories to recognize table tennis motions, propose a varying-length-input CNN framework, and compare the results with those of traditional recognition algorithms. The comparison results are shown in Table 4.

**Table 4:** The performance of the six algorithms on the TTMD6 dataset.

No	Algorithm	Accuracy
1	Varying-length-input CNN(paddle)	99.78%
2	Standard CNN(paddle)^30^	97.66%
3	Standard CNN(joints)^30^	96.4%
4	LSTM^31^	76.6%
5	FNN^32^	98.4%
6	Athlete recognition	92.63%

We use six algorithms to recognize table tennis motions in the TTMD dataset.

**Figure 5:**
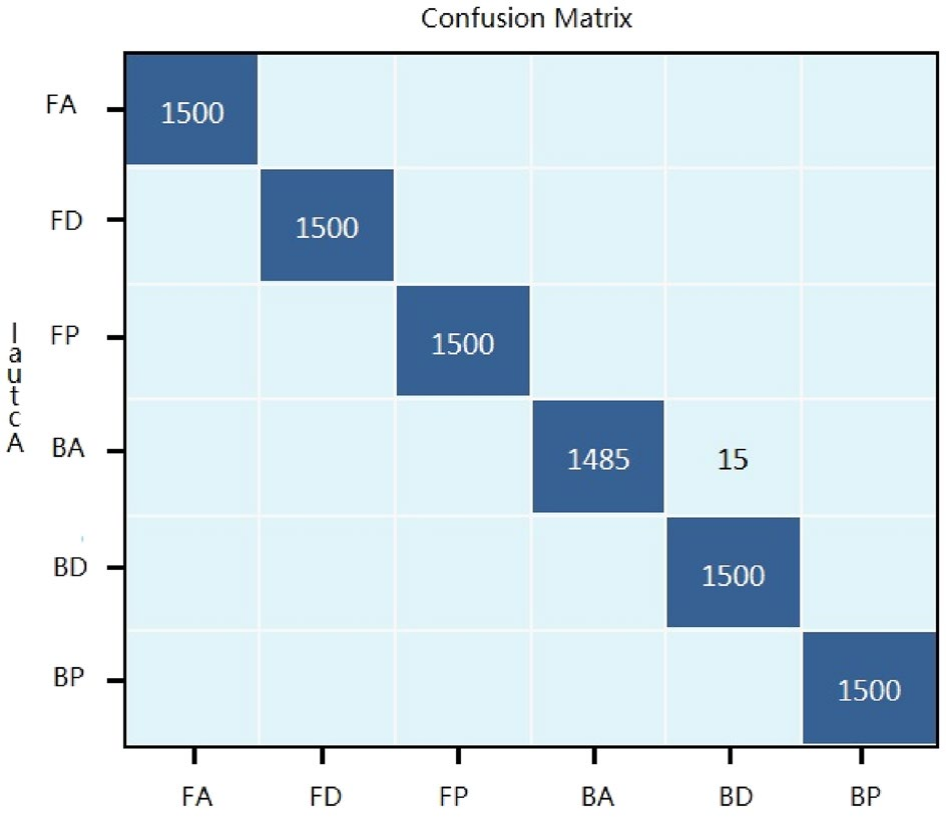
Confusion Matrix of the varying-length-input convolution neural networks.

The accuracy of our proposed algorithm in recognizing motions through paddle trajectories is 99.78%, which is 2.12% greater than that of standard three-layer CNNs. The confusion matrix is shown in Fig. 5. The LSTM algorithm accuracy in recognizing motions through the paddle trajectory is 76.6%. The accuracy of the FNN algorithm in recognizing motions through paddle trajectories is 98.4%. The accuracy of professional athletes in recognizing motions through paddle trajectories is 92.63%. The accuracy of our proposed algorithm is 99.78%, which is much greater than that of athletes. Both the dataset and the recognition algorithms in this paper are available for download.

## Conclusions

The results of this study showed that the TTMD6 dataset that we created is stable and can be used for research on table tennis motion recognition and biomechanics. We obtained a recognition accuracy of 92.63% by using the paddle trajectory to create an animation and recruiting 40 professional table tennis athletes for motion recognition, indicating that it is feasible to use the paddle trajectory to recognize table tennis motions. We propose a varying-length-input CNN model, use our model to recognize the racket trajectory time series and obtain a recognition accuracy of 99.78%, which is higher than that of traditional recognition algorithms and athlete recognition. The trained model can be used in auxiliary training systems, table tennis robots, motion-sensing games, virtual reality and other fields.

### Supplementary Information

Supplementary Information.

## Data Availability

Data can be obtained from the URL: https://pan.baidu.com/s/1lDdqULp6gsBovY7BaUJj9Q?pwd=epsk
